# Characterization of Receptor Binding Profiles of Influenza A Viruses Using An Ellipsometry-Based Label-Free Glycan Microarray Assay Platform

**DOI:** 10.3390/biom5031480

**Published:** 2015-07-16

**Authors:** Yiyan Fei, Yung-Shin Sun, Yanhong Li, Hai Yu, Kam Lau, James P. Landry, Zeng Luo, Nicole Baumgarth, Xi Chen, Xiangdong Zhu

**Affiliations:** 1 Key Laboratory of Micro and Nano Photonic Structures (Ministry of Education), Department of Optical Science and Engineering, Shanghai Engineering Research Center of Ultra-Precision Optical Manufacturing, Fudan University, 220 Handan Road, Shanghai 200433, China; E-Mail: fyy@fudan.edu.cn; 2 Department of Physics, University of California, Davis, CA 95616, USA; E-Mails: bacan1125@gmail.com (Y.-S.S.); jim.landry@gmail.com (J.P.L.); 3 Department of Physics, Fu-Jen Catholic University, New Taipei City 24205, Taiwan; 4 Department of Chemistry, University of California, Davis, CA 95616, USA; E-Mails: yhzli@ucdavis.edu (Y.L.); hyu@ucdavis.edu (H.Y.); edlau@ucdavis.edu (K.L.); chen@chem.ucdavis.edu (X.C.); 5 Center for Comparative Medicine, University of California, Davis, CA 95616, USA; E-Mails: zluo@ucdavis.edu (Z.L.); nbaumgarth@ucdavis.edu (N.B.)

**Keywords:** influenza A virus, glycans, binding profile, microarray, label-free, ellipsometry, biosensors, high-throughput, reaction kinetics

## Abstract

A key step leading to influenza viral infection is the highly specific binding of a viral spike protein, hemagglutinin (HA), with an extracellular glycan receptor of a host cell. Detailed and timely characterization of virus-receptor binding profiles may be used to evaluate and track the pandemic potential of an influenza virus strain. We demonstrate a label-free glycan microarray assay platform for acquiring influenza virus binding profiles against a wide variety of glycan receptors. By immobilizing biotinylated receptors on a streptavidin-functionalized solid surface, we measured binding curves of five influenza A virus strains with 24 glycans of diverse structures and used the apparent equilibrium dissociation constants (avidity constants, 10–100 pM) as characterizing parameters of viral receptor profiles. Furthermore by measuring binding kinetic constants of solution-phase glycans to immobilized viruses, we confirmed that the glycan-HA affinity constant is in the range of 10 mM and the reaction is enthalpy-driven.

## 1. Introduction

Human populations are continuously at risk of infection from influenza A viruses carried by many species as well as infected humans. Annually arrived influenza epidemics are considerable health and economic burdens on the society. Occasional pandemics have claimed large numbers of human lives in the past [1,2]. Influenza A viruses are categorized according to antigenic properties of their surface glycoproteins: hemagglutinin (HA, with 18 subtypes) and neuraminidase (NA, with 11 subtypes) [3]. Yet, such a coarse categorization reveals little about the pathogenicity and pandemic potential of a virus strain. Avian and swine influenza viruses frequently infect humans and yet rarely become pandemic in a human population. However, the few strains that managed to acquire the capacity to efficiently spread from human to human and subsequently became pandemics resulted in major losses of human lives over the last century. They include H1N1 strains in 1918, H2N2 in 1957, H3N2 in 1968, and H1N1 in 2009 [4,5,6]. Recent laboratory studies of mutations in highly pathogenic avian influenza (HPAI) H5N1 strains and novel avian influenza H7N9 strains raise grave concerns that these strains may sooner or later bring another, perhaps far more devastating pandemic to human populations [7,8]. Better understanding and characterization of molecular determinants of influenza viruses that are required for human-to-human transmission will provide the basis for assessment of pandemic potentials of early identified influenza virus strains. The recent mutation studies such as ones reported by Foucier *et al.* and Kawaoke *et al.* [9,10] are most valuable and at the same time controversial. These studies have led to changes in US policy regarding working with gain-of-function studies on highly pathogenic viruses. It is clear that *in-silico* experiments must be performed whenever possible to minimize these studies of high risks.

The receptor specificity of an influenza virus is determined by viral HA glycoprotein recognition of particular linkages of terminal sialic acids on glycan receptors of a host. The binding specificity of HA is considered a significant barrier for human-to-human transmission of a virus strain. A virus strain must adapt to α2-6-linked sialyl receptors in the upper respiratory tract of humans in order to propagate efficiently from human-to-human [11,12,13,14]. Avian virus strains preferentially bind to α2-3-linked sialyl glycans, human virus strains mainly recognize α2-6-linked sialyl glycans, while swine viruses can be captured by both α2-6-linked and α2-3-linked glycans [15,16,17]. Switching from α2-3-linkage recognition to α2-6-linkage recognition through mutation and reassortment is often the key step for an avian strain to become transmissive in a human population (H9N2 strains are an exception as they show affinity to both α2-6-linked and α2-3-linked glycans and can infect humans, and yet they are so far not adapted to human hosts). It is thus vital to monitor changes in the receptor specificity profile of circulating influenza virus strains and identify telltale signatures that forecast pandemics and in turn to allow timely development of vaccines.

Several methods are routinely used to characterize the receptor specificity profile of HA glycoproteins, including hemagglutination assays, solid-phase binding assays, and glycan microarray-based assays. The hemagglutination assay is one of the earliest methods for assessing influenza virus binding specificity to human red blood cells (RBCs) that are modified to express either α2-3-linked or α2-6-linked receptors on the cell surface [15,16,18]. More recent solid-phase assays evaluate binding profiles of influenza viruses to synthetic sialyl glycans in a glass-bottomed microplate. Captured glycans or viruses are either directly labeled or subsequently reacted with labeled secondary antibodies [19,20,21]. The main disadvantage of these two types of assays is their relatively low throughput. Glycan microarray-based assays enable evaluation of influenza virus binding reactions to a large pool of glycans in a single experiment [12,17,22,23]. The endpoints of the binding reactions on glycan microarrays are typically detected by either labeling viruses before or having the captured viruses reacting with secondary labeled probes afterwards [24,25]. There are a number of drawbacks of fluorescence-based glycan microarray detection: (1) labeling viruses can alter receptor specificity profiles; (2) endpoint measurements can depend on incubation time and post-incubation washing before fluorescence detection, for example only those binding reactions that occur significantly during incubation and also survive washing treatment are recorded; (3) valuable kinetic and thermodynamic information on virus-glycan binding reactions are not available. It is noteworthy that low affinity reactions do not necessarily mean low concentrations of relevant constituents in signaling pathways.

In this report, we demonstrate a *label-free* glycan-microarray-based assay platform detected with a scanning ellipsometry sensor [26,27,28,29,30]. The ellipsometry sensor measures changes in phase and amplitude of an illuminating optical beam *in situ* when surface-bound glycans capture viruses from the solution or surface-bound viruses capture glycans from the solution. It enables acquisition of binding curves (reaction kinetics) as well as endpoints of virus-glycan binding reactions. Other label-free biosensors using surface-bound glycans as receptors such as electrochemical impedance spectrometry (EIS) and field-effect transistor (FET) sensors have also been developed and demonstrated for detecting glycan-binding proteins and viruses. These techniques have significantly higher sensitivities and yet lower throughput and higher assay cost when compared with the present ellipsometry-based sensor [31,32]. The combination of high-throughput and reaction kinetic detection makes this platform potentially useful for tracking receptor specificity profiles of influenza viruses and in screening compounds for ligands that interfere with the virus-glycan binding.

## 2. Materials and Methods

### 2.1. Virus Propagation

Five human influenza A virus strains were studied in this work: A/Puerto Rico/8/1934 (A/PR8, H1N1), A/Kawasaki/173/2001 (A/K173, H1N1), A/Memphis/1971 (A/Mem71, H3N1), A/Udorn/307/1972 (A/Udorn72, H3N2), and A/Philippines/2/82/X-79 (A/Philips, H3N2). A/Mem71 is a reassortant strain carrying HA of A/Memphis/1/71 (H3) and NA of A/Bellamy/42 (N1). For amplification of A/PR8, A/Mem71, A/Udorn72, and A/Philips, fertilized hen eggs were incubated for 10 days with constant rotation at 37 °C and 65% humidity. Eggs containing live embryos were infected with predetermined optimal concentrations of influenza virus strains by inoculation into the allantoic cavity and incubated for 2 days at 35 °C followed by overnight incubation at 4 °C. Allantoic fluid was harvested, batched, and centrifuged (8000× *g*, 15 min at 4 °C). Supernatants were divided into aliquots, snap-frozen on dry ice and stored at −80 °C until use. A/K173 strain was only amplified in MDCK cells as follows to avoid the chance of acquiring α2-3-linkage recognition due to passage through chicken eggs. MDCK cells were seeded at 3 × 10^5^ cells/mL in a 6-well plate and incubated at 37 °C overnight. Various concentrations of A/Kawasaki/173/2001 (A/K173, H1N1) virus were added to the washed cells and incubated for 45 min at 37 °C before 2 mL medium was added to each well. After 72 h, supernatants were taken, centrifuged at 500× *g*, 15 min at 4 °C, aliquoted, snap-frozen on dry ice and stored at −80 °C until use.

### 2.2. Virus Purification

Virus particles were precipitated from supernatants by overnight incubation at 4 °C with 8% polyethylene glycol 6000 solution (Sigma-Aldrich, Dallas, TX, USA) and pelleted by centrifugation (12,000 g, 30 min at 4 °C). The pellet was then resuspended in phosphate buffered saline (PBS), sonicated, and centrifuged for 5 min at 3000 g to release the viruses. The supernatant was collected and centrifuged (24,000 g, 2 h at 4 °C). The virus pellets were resuspended in a small volume of 1 × PBS and separated by sucrose gradient centrifugation (linear gradient 70%–25%, 24,000× *g*, 2 h at 4 °C). The virus band was harvested, resuspended in 1 × PBS and centrifuged (24,000× *g*, 2 h at 4 °C). The pellet containing the viruses was stored in a small volume of 1 × PBS at 4 °C. The virus concentration was determined by standard hemagglutination assays using chicken red blood cells [33]. The virus stock concentration for A/PR8, A/K173, A/Mem71, A/Udorn72, A/Philips is 2.5 × 10^6^ HAU/mL, 1 × 10^3^ HAU/mL, 2.5 × 10^6^ HAU/mL, 1.0 × 10^6^ HAU/mL and 0.5 × 10^6^ HAU/mL, respectively. We note that purification of viruses was done only to ensure that our study was not affected by non-specific reactions. Furthermore the sample needed can be significantly reduced at the expense of assay time.

### 2.3. Synthesis of Biotinylated Glycans

Table 1 lists 24 synthetic glycans (oligosaccharides) used in the present study. They include four β1-4-linked galactosides, three β1-3-linked galactosides, one β-linked galactoside, one α-linked *N*-acetylgalactosaminide, eight α2-3-linked sialosides, and seven α2-6-linked sialosides. The synthesis of these compounds was reported previously [29]. Briefly, chemoenzymatic synthesis of sialosides was achieved using a one-pot three-enzyme system [34,35,36]. In this system, *N*-acetylmannosamine (ManNAc), *N*-glycolylmannosamine (ManNGc), or mannose was converted to *N*-acetylneuraminic acid (Neu5Ac), *N*-acetylneuraminic acid (Neu5Gc), or 2-keto-3-deoxy-D-glycero-D-galactonononic acid (KDN) by an aldol condensation reaction catalyzed by an *Escherichia coli* (*E. coli*) K-12 sialic acid aldolase, and then activated to form CMP-sialic acids catalyzed by a *Neisseria meningitidis* CMP-sialic acid synthetase (NmCSS) [37]. The sialic acid residue in CMP-sialic acid was then transferred to propyl azide-tagged galactose-terminated acceptors by a multifunctional *Pasteurella multocida* sialyltransferase 1 (PmST1) [35] to form α2-3-linked sialosides, or by a *Photobacterium damselae* α2-6-sialyltransferase (Pd2-6ST) [36] to form α2-6-linked sialosides. Sialoside products were purified by Bio-Gel P-2 gel filtration chromatography, and the structures were characterized by ^1^H and ^13^C NMR as well as mass spectrometry. The azido group in the glycan products were reduced to an amino group and coupled to *N*-hydroxyl succinamide (NHS)-activated hexa(ethylene glycol) (HEG)-linked biotin as reported previously [38].

**Table 1:** Twenty-four biotinylated oligosaccharides and assigned identification numbers used in the present work.

Glycan I.D.	Glycan Structures				
OS-1		Gal	β-Biotin		
OS-2		GalNAc	α-Biotin		
OS-3		Gal	β1-4Glc	β-Biotin	
OS-4		Gal6S	β1-4Glc	β-Biotin	
OS-5		Gal	β1-4GlcNAc	β-Biotin	
OS-6		Gal	β1-4GlcNAc6S	β-Biotin	
OS-7		Gal	β1-3GlcNAc	β-Biotin	
OS-8		Gal	β1-3GlcNAc	β1-3Galβ1-4Glc	β-Biotin
OS-9		Gal	β1-3GalNAc	β-Biotin	
OS-10	Neu5Acα2-3	Gal	β-Biotin		
OS-11	Neu5Acα2-3	Gal	β1-4Glc	β-Biotin	
OS-12	Neu5Acα2-3	Gal6S	β1-4Glc	β-Biotin	
OS-13	Neu5Acα2-3	Gal	β1-4GlcNAc	β-Biotin	
OS-14	Neu5Acα2-3	Gal	β1-4GlcNAc6S	β-Biotin	
OS-15	Neu5Acα2-3	Gal	β1-3GlcNAc	β-Biotin	
OS-16	Neu5Acα2-3	Gal	β1-3GlcNAc	β1-3Galβ1-4Glc	β-Biotin
OS-17	Neu5Acα2-3	Gal	β1-3GalNAc	β-Biotin	
OS-18	Neu5Acα2-6	GalNAc	α-Biotin		
OS-19	Kdnα2-6	Gal	β1-4Glc	β-Biotin	
OS-20	Neu5Gcα2-6	Gal	β1-4Glc	β-Biotin	
OS-21	Neu5Acα2-6	Gal	β1-4Glc	β-Biotin	
OS-22	Neu5Acα2-6	Gal	β1-4GlcNAc	β-Biotin	
OS-23	Neu5Acα2-6	Gal	β1-4GlcNAc6S	β-Biotin	
OS-24	Neu5Acα2-6	Gal	β1-3GlcNAc	β-Biotin	

### 2.4. Fabrication of Glycan Microarrays

Twenty-four synthesized glycans were separately dissolved in 1 × PBS to 50 μM for microarray fabrication. Using an OmniGrid 100 contact-printing robot (Digilab, Holliston, MA, USA), six microarrays were printed on a streptavidin-functionalized glass slide. Each microarray contains 96 spots that consist of four replicates of 24 biotinylated glycans. The average diameter of printed spots is 130 µm and the center-to-center spot separation is 250 µm. The printed slides were stored at –20 °C for at least 24 h before use. According to the vendor (ArrayIt, Sunnyvale, CA), the surface density of streptavidin tetramer is ~1 × 10^12^/cm^2^. Assuming that each tetramer makes available two binding pockets to biotinylated glycans, the surface density of immobilized glycans is estimated to be roughly 2 × 10^12^/cm^2^, two orders of magnitude larger than the density of a full layer of influenza virus (~1 × 10^1^^0^/cm^2^). This means that subsequent binding of influenza viruses to these glycan microarrays is subject to the multi-valent (avidity) effect.

### 2.5. Fabrication of Influenza Virus Microarrays for Monovalent Glycan-HA Affinity Assays

To evaluate the monovalent glycan-HA binding affinity, we fabricated influenza virus microarrays from printed glycan microarrays as follows. Three biotinylated glycans (OS-22, OS-23, and OS-24) were printed into one-dimensional arrays on a streptavidin-coated glass slide. Each one-dimensional (1D) microarray consists of four replicates of OS-22, OS-23, and OS-24. We incubated the microarrays in an influenza virus solution in 1 × PBS at 10^4^ HAU/mL mixed with 0.1 mM NA inhibitor Zanamivir (AmplaChem and Labs, Carmel, IN, USA) until a full layer of viruses was captured by the glycans. Due to the avidity effect, the captured viruses do not dissociate from the surface for many hours. The HA glycoproteins on the far side of the captured viruses are accessible to solution-phase glycans in subsequent binding reactions.

### 2.6. Virus Binding Assay on Glycan Microarrays and Label-free Detection

A printed glass slide is assembled with a variable-temperature fluidic system with six chambers, each containing one of the six glycan microarrays [39]. The microarray-covered surface is washed with 1× PBS to remove excess printed materials and blocked with 0.5 mg/mL biotin-conjugated bovine serum albumin (Vector Laboratories, Burlingame, CA, USA) to prevent non-specific binding in subsequent assays. Viruses of a particular strain are diluted to 10^3^–10^4^ HAU/mL in 1 × PBS containing 0.1 mM NA inhibitor Zanamivir (AmplaChem and Labs, Carmel, IN, USA). As one HAU typically corresponds to 10^6^ viral particles [40], the concentration of viruses is between 10^12^–10^13^/L, or 10^−11^–10^−1^^0^ M. With roughly 1000 HA glycoproteins per virus particle [41], the HA concentration is in the range of 10^−8^–10^−7^ M (10^15^–10^16^ spikes/L). Since a hemagglutination assay measures the amount of viruses needed to agglutinate a fixed quantity of red blood cells, the assay and in turn HAU are related to the avidity of HA on the virus to glycan receptors on red blood cells instead of the affinity of HA to glycan receptors. If a strain of influenza A virus agglutinates red blood cells with smaller avidity (larger equilibrium dissociation constant), one HAU of such a strain will correspond to a larger number of viral particles and vice versa. To acquire virus-glycan association-dissociation curves, we pass 0.17 mL of a virus solution through a chamber at a rate of 2.0 mL/min to replace 1× PBS to start the association phase. We then reduce the flow rate to 0.01 mL/min during the remainder of the association phase. At the end of the association phase, we pass 0.5 mL of 1 × PBS through the chamber at 2.0 mL/min to replace the virus solution and then reduce the flow rate to 0.01 mL/ min for the dissociation phase of the reaction.

We measured the amount of influenza viruses captured by surface-bound glycans with a scanning ellipsometry sensor (a.k.a. oblique-incidence reflectivity difference or OI-RD scanning microscope) [26,27,28,29,30]. As illustrated in Equation (5) of Reference #30, the optical sensor signal is proportional to the surface mass density (the product of the volume mass density and the thickness) of captured viruses. During association and dissociation phases of the reaction, we read out sensor signals from 96 targets and an equal number of references (adjacent to the targets) every 2 s. The reference signals are used to remove the drift in the sensor system.

## 3. Results

### 3.1. Binding Curves of Influenza A Virus to Glycan Microarrays

**Figure 1:**
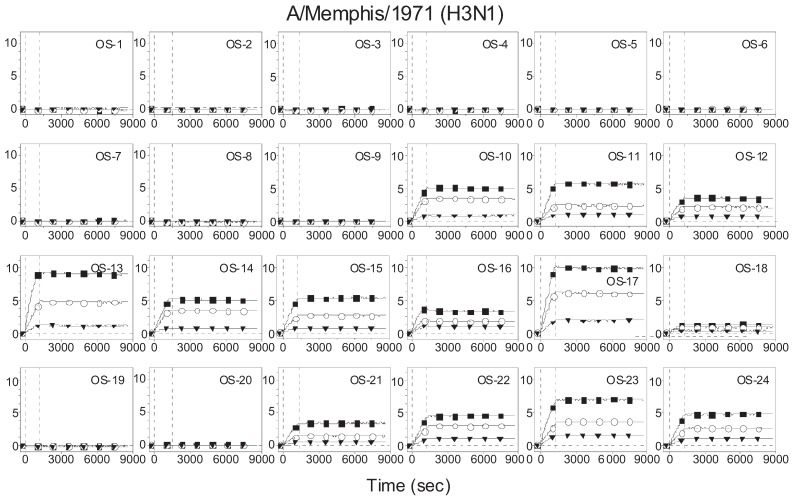
Association-dissociation (binding) curves of A/Mem71 to 24 biotinylated oligosaccharides immobilized in microarray format on streptavidin functionalized glass slide. The y-axis displays the ellipsometry signal in arbitrary unit and is proportional to the surface mass density of the capture viruses. An ellipsometry signal of 20 corresponds to a fully covered monolayer of influenza A viruses. The viral concentrations are 2.5 × 10^4^ HAU/mL (solid squares), 1.25 × 10^4^ HAU/mL (open circles), and 0.42 × 10^4^ HAU/mL (solid triangles). The virus solutions were mixed with 0.1 mM Zanamivir. Vertical lines mark starts of association and dissociation phases of the binding events, respectively. The solid lines through the curves are global fits to a 1-to-1 Langmuir reaction model to yield apparent equilibrium dissociation constant.

Figure 1 displays association-dissociation curves of A/Mem71 (H3N1) to surface-bound synthetic glycans. The ellipsometry sensor signal, displayed in arbitrary unit, is proportional to the surface mass density of the capture viruses. A signal of 20 corresponds to a fully covered monolayer of influenza A viruses. The virus binds to both α2-3-linked (OS-10 through OS-17) and α2-6-linked (OS-18 and OS-21 through OS-24) sialosides. It does not bind to glycans either lacking terminal sialic acids (OS-1 through OS-9) or having terminal but modified sialosides (OS-19 and OS-20). Dissociation of captured viruses is negligible over as long as 8 h. Such a remarkable stability of the virus-glycan complexes is due to the avidity effect—a single virus binding with multiple sialyl glycan receptors on the solid surface [42]. Avidity plays an important role in both virus infection and virus antigenic drift [43,44]. We use avidity constants extracted from the binding curves to characterize the receptor specificity of the virus. Such a receptor specificity profile can be compared with hemagglutinin assays as the latter are also avidity-based.

### 3.2. Receptor Specificity Profiles of Influenza Viruses of Subtype H3 and H1

**Figure 2:**
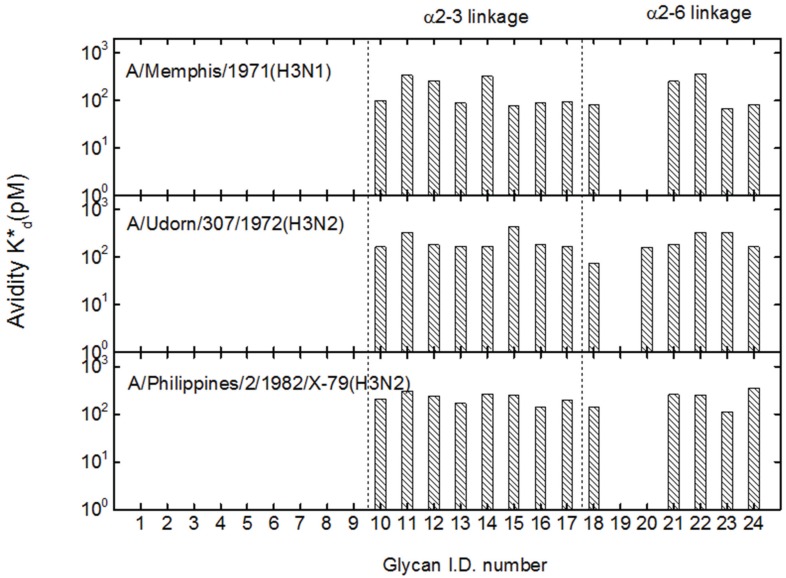
Avidity-enhanced equilibrium dissociation constants of three H3-subtype influenza viruses, A/Mem71 (top panel), A/Udorn72 (middle panel) and A/Philips (bottom panel), with 24 glycans designated by the I.D. numbers as listed in Table 1. OS-10 through OS-17 are α2-3-linked sialosides. OS-18 through OS-24 are α2-6-linked sialosides. A/Mem71, A/Udorn72 and A/Philips bind to both α2-3-linked and α2-6-linked sialosides.

We fit association-dissociation curves of A/Mem71 to a Langmuir reaction kinetic model with apparent association rates (k_on_^*^) and dissociation rates (k_off_^*^) as parameters. The corresponding apparent equilibrium dissociation constants are computed as K_d_^*^ = k_off_^*^/k_on_^*^. The latter are in the range of 100 pM. The top panel of Figure 2 displays K_d_^*^ for A/Mem71 to 24 synthetic glycans to represent the avidity binding profile. The bottom two panels of Figure 2 show avidity binding profiles of two other H3 virus strains (A/Udorn72 and A/Philips) to the same 24 glycans. All three strains bind to both α2-3-linked and α2-6-linked sialyl glycans with comparable equilibrium dissociation constants of 100 pM [17,45,46,47]. As a result, one HAU of these viruses has roughly the same number of viral particles. It is noteworthy that A/Udorn72 also binds to Neu5Gc-terminated sialic acid (OS-20), while A/Mem71 and A/Philips do not. Neu5Gc is a common sialic acid found in tissues of many mammals such as bovine, equine, and swine, while human tissues possess only slight concentrations of Neu5Gc (less than 0.1% of total sialic acids) [48].

**Figure 3:**
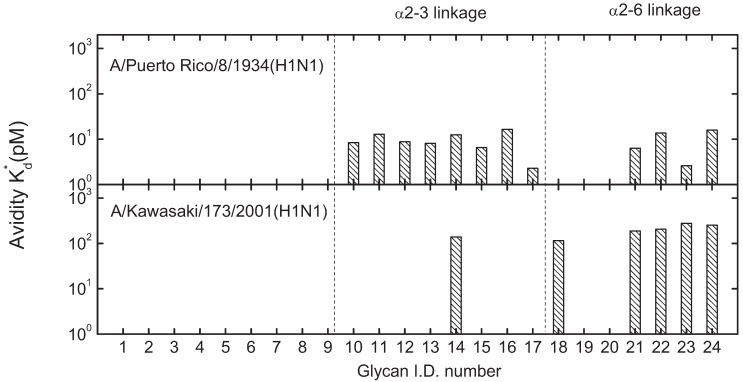
Avidity-enhanced equilibrium dissociation constants of two H1 influenza viruses, A/PR8 (top panel) and A/K173 (bottom panel), to the 24 glycans as listed in Table 1. A/PR8 binds to both α2-3- and α2-6-linked sialosides, while A/K173 preferentially recognizes α2-6-linked sialosides. A/PR8 clearly binds to the glycan receptors more strongly than A/K173, A/Mem71, A/Udorn72 and A/Philips.

Figure 3 shows the avidity binding profiles of two H1 virus strains. A/PR8 binds to both α2-3- and α2-6-linked sialyl glycans [15,49,50]. In contrast MDCK-grown A/K173 strain binds to all five α2-6-linked sialosides and to only one of eight α2-3-linked sialosides, exhibiting a clear preference for α2-6-linked sialic acids. The preference of binding to α2-6-linked sialosides by A/K173 has been observed previously [8,51]. The apparent equilibrium dissociation constants for A/PR8 are one order of magnitude smaller than those for A/K173 and the three H3 strains indicating that A/PR8 has a stronger avidity to the glycan receptors. This also means that one HAU of A/PR8 has a smaller number of viral particles than there are in one HAU of the other four strains, and therefore the actual K_d_^*^ for A/PR8 is even smaller.

### 3.3. Mono-valent Equilibrium Dissociation Constants (Affinity Constants) and Thermodynamics of Sialyl Glycan-HA Reactions

**Figure 4:**
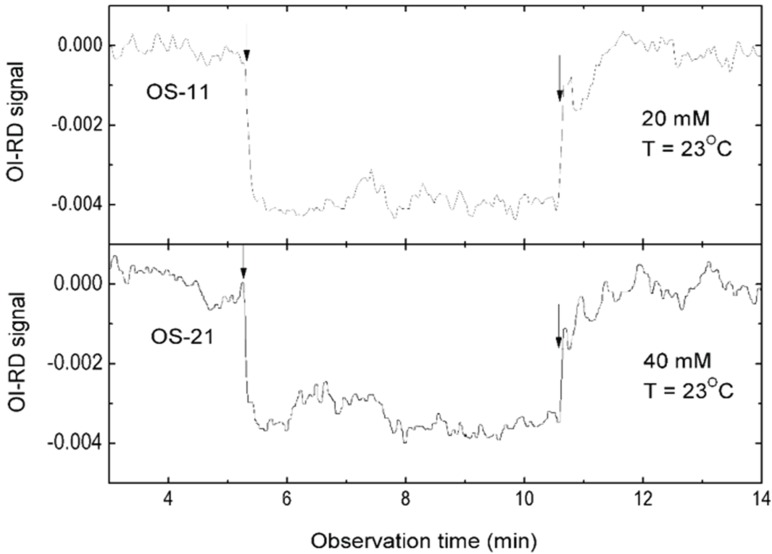
Association-dissociation curves of solution-phase α2-3-linked sialyllactose (OS-11 without the biotin linker, 20 mM) and α2-6-linked sialyllactose (OS-21 without the biotin linker, 40 mM) to immobilized A/Mem71 on the solid surface, acquired at 23 °C (T = 296 K).

To study glycan-HA binding reactions without the avidity effect, we repeated the reactions for two sialyl glycans in solution with virus microarrays. In this case, each binding pocket on a HA homo-trimeric glycoprotein on the virus surface reacts independently with solution-phase sialyl glycans and the equilibrium dissociation constant is of a monovalent eaction. For glycan solutions, we dilute α2-3-sialyllactose (OS-11 without the linker) and α2-6-sialyllactose (OS-21 without the linker) (Carbosynth Limited, Berkshire, UK) solutions in 1× PBS to concentrations of 20–40 mM. To capture fast reaction kinetics, we read out sensor signals from 12 immobilized virus targets and 12 references every 60 ms. Figure 4 shows association-dissociation curves of α2-3-sialyllactose and α2-6-sialyllactose (at 20 mM and 40 mM respectively) to immobilized A/Mem71 strains at T = 296K. Unlike association of viral particles or bulky proteins with immobilized glycans that mostly adds a layer of protein mass to the surface and in turn causes the sensor signal to increase, the capture of small glycan molecules by HA on the viral surface changes the effective refractive index of HA that in turn causes the sensor signal to decrease instead [52]. It is clear from Figure 4 that dissociation rates of sialyl glycans from immobilized A/Mem71 are at least five orders of magnitude larger than dissociation rates of A/Mem71 from the same but immobilized sialyl glycans (Figure 1). This confirms that the stability of captured viruses to high density surface-bound sialyl glycans is the result of multi-valence or avidity effect. To determine mono-valent equilibrium dissociation constants (affinity constant) and thermodynamics of glycan-HA reactions, we measured association-dissociation curves of α2-3-sialyllactose and α2-6-sialyllactose with immobilized A/Mem71 strans at four glycan concentrations and four temperatures between 288 K and 298 K. We globally fit the curves to the Langmuir reaction model to find the monovalent association rate constants k_on_(T) and dissociation rate constants k_off_(T). We compute the equilibrium dissociation constants K_d_(T) = k_off_(T)/k_on_(T). The results are summarized in Table 2. K_d_(T) are of the order of mM, consistent with nuclear magnetic resonance (NMR) studies reported by others [13,53,54]. If one homo-trimeric HA binds to three sialyl glycans on the solid surface, each with a mono-valent equilibrium dissociation constant of mM, the resultant apparent equilibrium dissociation constant is easily reduced to 100 pM or less as shown in Figure 2. It means that one or two homo-trimeric HA glycoproteins on the viral surface are sufficient to stabilize a virus-glycan complex with apparent equilibrium dissociation constants in the range of 10–100 pM. We note that monovalent equilibrium dissociation constants for reactions of HA with α2-3-linked and α2-6-linked sialyllactoses are comparable [23].

From the temperature dependence of K_d_(T) from 288 K to 298 K, we extracted ΔG = ΔH − TΔS (Gibbs free energy change), ΔH (enthalpy change), and ΔS (entropy change) for the HA-sialyllactose reactions [39]. The results are summarized in Table 3. The reactions of HA of A/Mem71 with the two sialyllactoses are driven by a large enthalpy reduction (ΔH~−32 to −41 kcal/mol), balanced by a significant entropy loss (TΔS~−30 to −39 kcal/mol) [55].

**Table 2:** Equilibrium rate constants of monovalent sialyllactoses (OS-11 and OS-21) binding to hemagglutinin (HA) on immobilized influenza virus A/Mem71 (H3N1) and apparent equilibrium rate constants of multivalent binding of HA on influenza virus A/Mem71 to immobilized sialyllactoses at T = 298K.

Glycans	Kinetics constants			Apparent kinetics constants		
	K_on_ (1/M·s)	K_off_ (1/s)	K_d_ (mM)	K^*^_on_ (1/M·s)	K^*^_off_ (1/s)	K^*^_d_ (mM)
α2-3 (OS-11)	2.8	7.36 × 10^−2^	26.3	7.2 × 10^3^	4.1 × 10^−6^	5.8 × 10^−7^
α2-6 (OS-21)	2.7	1.28 × 10^−1^	47.6	7.0 × 10^3^	<3.0 × 10^−6^	<4.3 × 10^−7^

**Table 3:** Equilibrium dissociation constants of A/Mem71with α2-3-sialyllactose (OS-11) and α2-6-sialyllactose (OS-21) between 288K and 298K, and the corresponding changes in thermodynamic functions as a result of complex formation as deduced from the temperature dependence of the dissociation constants.

Glycans	Temperature (K)	K_d_ (mM)	ΔG (kcal/mol)	ΔH (kcal/mol)	ΔS (kcal/mol·K)	TΔS (kcal/mol)
α2-3 sialyllactose (OS-11)	288	2.6	−3.44	−41.4	−0.13	−37.9
	293	6.4	−2.96	−41.4	−0.13	−38.6
	296	18.8	−2.35	−41.4	−0.13	−39.0
	298	26.3	−2.16	−41.4	−0.13	−39.2
α2-6 sialyllactose (OS-21)	288	6.8	−2.87	−32.2	−0.10	−29.2
	293	11.2	−2.63	−32.2	−0.10	−29.7
	296	23.8	−2.21	−32.2	−0.10	−30.0
	298	47.6	−1.82	−32.2	−0.10	−30.2

## 4. Discussion and Conclusions

We demonstrated a microarray-based label-free sensor for characterizing virus-glycan binding profiles. By immobilizing biotinylated glycan receptors on streptavidin-coated surface that exposes terminal sialyl residues and penultimate glycan structures, this platform yields same receptor specificity profiles for influenza viruses as those obtained by hemagglutination assays. The advantages over hemagglutination assays are label-free detection, high-throughput, and availability of kinetic and thermodynamic information on glycan-virus binding reactions. Such a platform can be used for surveillance of receptor specificity changes for influenza viruses such as H9N2, H5N1 or H7N9 avian influenza strains, and for screening of synthetic and natural compounds that interfere with virus-receptor binding reactions [26]. For example, it is known that HA of the H9N2 strain has already acquired a mutation predominantly found in human-adapted viruses and it is thus particularly relevant to monitor the receptor specificity profile of this virus strain for changes that may lead to possible pandemics. Furthermore for naturally occurring viruses, this platform can be used to determine whether changes in amino acids involved in receptor binding indeed change receptor profiles. For these purposes, microarrays of a more comprehensive collection of glycans than the present 24 glycans are required for virus finger-printing and receptor profiling. Our present platform can easily detect reactions of a single virus stain with up to 13,000 immobilized glycans in a single experiment. The real challenge is the access to a structurally diverse and comprehensive collection of glycans suitable for virus receptor profiling. Although glycan libraries exist in a number of academic institutions, they are not generally available.

For further insight into receptor specificity profiles, we note that all five human virus strains studied in this work, two H1N1 strains, one H3N1 strain and two H3N2 strains, recognize α2-6-linked sialyl glycans. A/Puerto Rico/8/1934 (A/PR8, H1N1), A/Memphis/1971 (A/Mem71, H3N1), A/Udorn/307/1972 (A/Udorn72, H3N2), and A/Philippines/2/82/X-79 (A/Philips, H3N2) also recognize α2-3-linked sialyl glycans with comparable equilibrium dissociation constants. Since these four strains had been passed through chicken eggs for an unknown number of times, their affinities to α2-3-linked sialyllactoses are likely acquired attributes typical of avian virus strains. A/K173 strain had only been passed through MDCK and shows a clear preference of binding to α2-6-linked sialosides.

Specific recognition of α2-3-linked and/or α2-6-linked glycan receptors by an influenza virus strain is linked to a number of key amino acid residues in and around the receptor binding site (RBS) on the HA glycoprotein. RBS consists of three secondary structures: the 190 helix (residues 190–198), the 130 loop (residues 135–138), and the 220 loop (residues 221–228) [56]. Mutations within the RBS can induce substantial changes in receptor specificity. In Table 4, we list amino acid residues at five positions, 138/190/225/226/228, for 38 human H1 strains and 23 human H3 strains along with their receptor specificities. It is feasible to observe some useful rules as to which combination of these amino acid residues tend to be essential for certain receptor specificity: (1) For both H1 and H3 strains, the α2-3 specificity requires either simultaneous 138A/190E or 138A/190D/225G/226Q/228G(S); (2) For H1 strains, the α2-6 specificity requires simultaneous 225D(G)/226Q/228G; (3) For H3 strains, the α2-6 specificity requires simultaneous 225G/Q226L/228S.

**Table 4:** Five amino acid residues at the receptor binding site (RBS) of HA glycoproteins for 38 human H1 strains and 23 human H3 strains and their respective receptor specificity.

Virus strain	Subtype	138	190	225	226	228	α2-3	α2-6
A/Kawasaki/173/2001	H1	S	D	D	Q	G	−	+
A/Puerto Rico/8/1934	H1	A	E	D	Q	G	+	+
A/Memphis/1971	H3	A	E	G	L	S	+	+
A/Udorn/307/1972	H3	A	E	G	L	S	+	+
A/Philippines/2/1982/X-79	H3	T	E	G	L	S	+	+
A/Memphis/14/1996-M [22]	H1	S	D	D	Q	G	−	+
A/New Caledorial/20/1999 [57]	H1	S	D	D	Q	G	−	+
A/Oklahoma/447/2008 [58]	H1	S	D	D	Q	G	−	+
A/Ohio/07/2009 [57]	H1	A	D	D	Q	G	−	+
A/Texas/05/2009 [57]	H1	A	D	D	Q	G	−	+
A/NewYork/18/2009 [57]	H1	A	D	D	Q	G	−	+
A/South Carolina/1/1918 [59]	H1	A	D	D	Q	G	−	+
A/South Carolina/1/1918 [12]	H1	A	D	D	Q	G	−	+
A/South Carolina/1/1918 [56]	H1	A	D	D	Q	G	−	+
A/South Carolina/1/1918 (D225G) [56]	H1	A	D	G	Q	G	+	+
A/California/4/2009 [56]	H1	A	D	D	Q	G	−	+
A/California/4/2009 (D225E) [56]	H1	A	D	E	Q	G	−	+
A/California/4/2009 (D225G) [56]	H1	A	D	G	Q	G	+	+
A/California/4/2009 [22]	H1	A	D	D	Q	G	+	+
A/California/07/2009 [23]	H1	A	D	D	Q	G	+	+
A/California/07/2009 [60]	H1	A	D	D	Q	G	−	+
A/Brisbane/59/2007 [23]	H1	S	N	D	Q	G	+	+
A/Hamburg/5/2009 [22]	H1	A	D	D	Q	G	+	+
A/Iowa/1/2006 [22]	H1	A	D	N	Q	G	+	+
A/Mexico/Indre/4114/2009 [57]	H1	A	D	G	Q	G	+	+
A/New Jersey/1976 [22]	H1	A	D	G	Q	G	+	+
A/New Jersey/1976 [57]	H1	A	D	G	Q	G	+	+
A/New York/1/1918 [59]	H1	A	D	G	Q	G	+	+
A/New York/1/1918 [12]	H1	A	D	G	Q	G	+	+
A/New York/1/1918(D190E) [12]	H1	A	E	G	Q	G	+	−
A/New York/4/2009 [57]	H1	A	D	G	Q	G	+	+
A/Texas/36/1991 [12]	H1	S	D	D	Q	G	+	+
A/Puerto Rico/8/1934 [50]	H1	A	E	D	Q	G	+	+
A/Puerto Rico/8/1934 [15]	H1	A	E	D	Q	G	+	+
A/Fort Monmouth/1/1947 [15]	H1	A	D	G	Q	G	+	+
A/Roma/1/1949 [15]	H1	A	D	G	Q	G	+	+
A/Malaya/302/1954 [15]	H1	A	D	G	Q	G	−	+
A/Denver/1957 [15]	H1	A	E	D	Q	G	+	−
A/New Jersey /8/1976 [15]	H1	A	D	G	Q	G	−	+
A/USSR/90/1977 [15]	H1	S	D	G	Q	G	−	+
A/Brazil/11/1978 [15]	H1	S	D	G	Q	G	−	+
A/India/6263/1980 [15]	H1	S	N	D	Q	G	−	+
A/Chile/1/1983 [15]	H1	A	D	N	Q	G	−	+
A/Taiwan/1/1986 [15]	H1	S	D	G	Q	G	−	+
A/Memphis/12/1986 [15]	H1	S	D	G	Q	G	−	+
A/CHR/157/1983 [15]	H1	S	D	D	Q	G	−	+
A/Kawasaki/173/2001 [51]	H1	S	D	D	Q	G	−	+
A/Kawasaki/173/2001 [8]	H1	S	D	D	Q	G	−	+
A/Aichi/2/1968 [46]	H3	A	E	G	L	S	+	+
A/Aichi/2/1968 [61]	H3	A	E	G	L	S	+	+
A/Memphis/102/1972 [46]	H3	A	E	G	L	S	+	+
A/LosAngeles/2/1987 [46]	H3	A	E	G	L	S	+	+
A/Udorn/307/1972 [61]	H3	A	E	G	L	S	+	+
A/Philippines/2/1982/X-79 [17]	H3	A	E	G	I	S	+	+
A/Victoria/3/1975 [61]	H3	A	E	G	L	S	+	+
A/Shanghai/11/1989 [46]	H3	A	E	G	L	S	+	+
A/Udorn/307/1972 [46]	H3	A	E	G	L	S	+	+
A/Udorn/307/1972 [17]	H3	A	E	G	L	S	+	+
A/Udorn/307/1972(E190D) [45]	H3	A	D	G	L	S	−	+
A/Hongkong/1/1968 [60]	H3	A	E	G	L	S	−	+
A/Texas/1/1977 [61]	H3	A	E	G	L	S	−	+
A/Shanghai/31/1980 [61]	H3	A	E	G	L	S	−	+
A/LosAngeles/2/1987 [61]	H3	A	E	G	L	S	−	+
A/Oklahoma/483/2008 [58]	H3	A	D	D	I	S	−	+
A/Oklahoma/323/2003 [17]	H3	A	D	D	I	S	−	+
A/Oklahoma/369/2005 [17]	H3	S	D	D	I	S	−	+
A/Oklahoma/1992/2005 [17]	H3	A	D	D	I	S	−	+
A/Tottori/872K4/1994 [62]	H3	A	D	G	L	S	−	+
A/Tottori/872AM2/1994 [62]	H3	A	D	G	L	S	−	+
A/Tottori/872AM4/1994 [62]	H3	A	D	G	Q	S	+	−
A/Tottori/872AM1AL3/1994 [62]	H3	A	D	G	Q	S	+	−
A/Tottori/872AM2AL3/1994 [62]	H3	A	D	G	Q	S	+	−
A/Mem/1/71-Bel42 [47]	H3	A	E	G	L	S	+	+

There are a number of exceptions and they raise important issues such as effects of *assay* methodology, additional passage history, and other amino acid residues. For *methodology-independent* exceptions such as A/Malaya/302/1954, A/Denver/1957 and A/New Jersey/8/1976, when examined with same hemagglutination assays, these H1 strains exhibit different specificity patterns even though they have presumably same amino acid residues at 138/190/225/226/228. In these cases the “exceptional” receptor specificity can stem from the influence of other amino acid residues or from different passage histories *after* the strains were initially isolated and sequenced. The same can be said of H3 strains such as A/Texas/1/1977, A/Shanghai/31/1980, and A/Los Angeles/2/1987 as they show no affinity to α2-3-linked sialoside even though they are presumably equipped with 138A/190E. A/Hongkong/1/1968 may belong to this exceptional group as well.

For *assay methodology-dependent* exceptions, Childs *et al.* [22] (using neoglycolipid (NGL)-functionalized solid surface for direct glycan immobilization) reported “exceptional” specificity profiles for A/California/4/2009, while Zhang *et al.* [56] (using streptavidin-functionalized solid surface for immobilization of biotinylated glycans) reported “normal” specificity profiles for the same virus strain. Liao *et al.* [23] (using NHS-activated solid surface for direct glycan immobilization) reported “exceptional” specificity profiles for A/California/07/2009 while Yang *et al.* [60] (using streptavidin-functionalized solid surface for immobilization of biotinylated glycans) reported “normal” receptor specificity for the same virus strain. Additionally Childs *et al.* [22] reported “exceptional” specificity to α2-3-linked sialosides for A/Hamburg/5/2009 and A/Iowa/1/2006 using neoglycolipid (NGL)-functionalized solid surface for direct glycan immobilization; Stevens *et al.* [12] reported “exceptional” specificity to α2-3-linked sialosides for A/Texas/36/1991 using NHS-activated solid surface for glycan immobilization. These inconsistent reports raise suspicions that “exceptional” receptor profiles in these cases may be attributed to NGL-based or NHS-based glycan immobilization chemistry which can present glycan receptors differently when compared to the streptavidin-based chemistry and need to be cross-checked with the findings from other validated assays.
